# Dorsal stream involvement in recognition of objects with transient onset but not with ramped onset

**DOI:** 10.1186/1744-9081-7-34

**Published:** 2011-08-16

**Authors:** Robin Laycock, Alana J Cross, Tomas Lourenco, Sheila G Crewther

**Affiliations:** 1 School of Psychological Science, La Trobe University, Melbourne, Australia

## Abstract

**Background:**

Although the ventral visual stream is understood to be responsible for object recognition, it has been proposed that the dorsal stream may contribute to object recognition by rapidly activating parietal attention mechanisms, prior to ventral stream object processing.

**Methods:**

To investigate the relative contribution of the dorsal visual stream to object recognition a group of tertiary students were divided into good and poor motion coherence groups and assessed on tasks classically assumed to rely on ventral stream processing. Participants were required to identify simple line drawings in two tasks, one where objects were presented abruptly for 50 ms followed by a white-noise mask, the other where contrast was linearly ramped on and off over 325 ms and replaced with a mask.

**Results:**

Although both groups only differed in motion coherence performance (a dorsal stream measure), the good motion coherence group showed superior contrast sensitivity for object recognition on the abrupt, but not the ramped presentation tasks.

**Conclusions:**

We propose that abrupt presentation of objects activated attention mechanisms fed by the dorsal stream, whereas the ramped presentation had reduced transience and thus did not activate dorsal attention mechanisms as well. The results suggest that rapid dorsal stream activation may be required to assist with ventral stream object processing.

## Background

It is now well established that most visual information projects through the Lateral Geniculate Nucleus (LGN) and primary visual cortex (V1) before dividing into two major cortical pathways [1-4]. Firstly, the dorsal stream, which is generally accepted to be responsible for motion perception, spatial awareness, and vision for action, includes areas V5, V3a, and V6, and terminates in parietal cortex. Secondly, the ventral stream, which is specialized for object recognition and includes areas such as the fusiform gyrus and the lateral occipital complex, terminates in temporal cortex [4-6].

These two visual streams are fed by differing ratios of magnocellular and parvocellular contributions, originating in different laminar layers of the LGN. The magnocellular pathway is predominantly involved in the processing of high temporal and low spatial frequencies at low luminance contrasts, and provides the great majority of the contribution to the dorsal stream. The subcortical parvocellular pathway on the other hand has been found to be optimally suited to the processing of colour and low temporal and high spatial frequencies at higher contrast. The parvocellular system mainly projects through to the ventral stream, though the magnocellular system also provides a substantial input to the ventral stream [2,7,8].

Many experiments have been devoted to understanding how the dorsal and ventral visual streams interact, contribute to conscious awareness of visual events, and the ability to make motor responses (e.g., eye- and hand- movements) [9-14].

One recent model of visual processing, which builds on the work of Bullier [15], proposes that fast subcortical projections of the magnocellular (M) pathway project through the dorsal stream to initiate exogenously-driven attention mechanisms in parietal and frontal cortex [16]. According to Bullier the earliest information to V5 is fed back into V1 in time for the initial parvocellular (P) arrivals in V1, and subsequent detailed or more local processing through the ventral stream. This conduction advantage of the M system over the P system into V1 (between 15 and 40 ms in humans) [17-19], leads to the model being termed the 'magnocellular advantage' [16]. A key aspect of this model predicts that rapid activation of frontoparietal attention mechanisms via V5 in the dorsal stream plays a preparatory and alerting role to a new salient visual event, and assists the fine-detailed processing of object features which occurs later in the temporal cortex, the termination point of the ventral stream.

The 'magnocellular advantage' model has been used to explain a range of investigations of visual processing. For example Laycock and Crewther [20] have argued that a reduction in the magnocellular latency advantage could interfere with the rapid activation of the parieto-frontal attention network and contribute to understanding the range of subcortical magnocellular, dorsal stream, and attentional deficiencies reported in developmental dyslexia [21-28]. Laycock et al. [13] have also shown that transcranial magnetic stimulation (TMS) of areas V1 and V5 in skilled readers leads to an impairment in word recognition at different times post word stimulus onset, arguing for a role for the dorsal stream in word processing.

Performance on global and local scene segmentation has also been associated with magnocellular pathway integrity [29]. Difficulty in identifying the global components of locally salient hierarchical Navon figures has been associated with higher rather than lower scores on the Autism Quotient (AQ). Sutherland and Crewther also showed that the initial cortical response of the magnocellular afferents was weaker for low contrast stimuli, and that the magnocellular but not the parvocellular pathway demonstrated a delayed response when stimuli were presented at high contrast, for high compared with low AQ participants. These findings were suggested to reflect support for impairment in the magnocellular feedback contributions normally apparent in the magnocellular advantage model [16].

A recent study by Levy, Walsh and Lavidor [30] compared two groups of skilled adult readers, subdivided by performance on a detection of motion coherence task. Most importantly for the current purposes, the good motion coherence group showed an advantage in classifying words as real words rather than nonwords. Despite all participants being skilled readers, motion coherence performance - a skill requiring dorsal stream processing, predicted rapid visual word identification, a presumed ventral stream task. These results were taken as support for the magnocellular advantage [16]. Levy et al. suggested that poor dorsal stream function may have reduced rapid attention activation in turn impeding the processing of letter strings through the ventral stream.

The current study's aim was to further investigate whether there is early dorsal stream involvement in abrupt onset object recognition. The magnocellular advantage model [16] predicts that abrupt presentation of salient stimuli will activate dorsal stream-driven exogenous attention, to facilitate object-specific ventral stream processing. Conversely, if the transient nature of the onset/offset of an object is removed - that is, if it does not appear abruptly and rather the onset is gradually ramped, then the rapid dorsal stream activation of attention mechanisms will not be activated, either at all, or perhaps as strongly or quickly. Ramped and abrupt onset object recognition stimuli have been used previously to argue that rapid perception requires transient component signalling [31]

We therefore compared a group of Good and Poor motion coherence detectors, created from a normal population of adults, on an abrupt and a non-abrupt (i.e., ramped) onset/offset match-to-sample object recognition task, utilising contrast as the dependent variable. Low contrast stimuli are expected to preferentially stimulate the sub-cortical magnocellular responses, but to require ventral stream processing for successful identification of objects. It was expected that good motion detectors would show superior contrast sensitivity for object recognition when contrast abruptly reaches its peak contrast when compared with poor motion detectors.

When the transient nature of the appearance of the object is removed by gradually increasing the object contrast in a linear fashion to a maximum contrast (ramped contrast onset) we predict such stimuli will not be well suited to activating the dorsal stream. Object presentation manipulated to purportedly reduce dorsal stream activation and parietofrontal attention mechanisms, was expected to not show any differences between good and poor motion coherence detectors.

## Methods

### Participants

Sixty-two university students participated in the experiment. Of these 6 were excluded (see Results section for details), giving a final sample of 56 (33 female). The mean age of participants was 22.39 (SD = 3.89), with a range of 19 to 34 years. All participants had normal or corrected to normal visual acuity, and gave their informed consent.

### Materials

Stimuli were presented on an eMac computer at a viewing distance of 57 cm. The monitor had an 80 Hz refresh rate, and tasks were programmed and presented using VPixx software (Version 2.4, http://www.vpixx.com). Participants also completed the Ravens Standard Progressive Matrices [32], which is a measure of non-verbal mentation whereby participants have 20 minutes to complete as many of the visual puzzles as possible in order to confirm that participants did not differ in general nonverbal intelligence.

#### Motion Coherence Detection Task

Two hundred white dots (5 pixels (0.17 deg at 57 cm) high and wide) were placed within an illusory square subtending 7.5° by 7.5° on a black background. A percentage of the dots moved coherently in one direction across the square whilst the remaining dots moved in random directions. All dots had a speed of 4 pixels per frame (11.3 deg/s at 57 cm), and a limited lifetime of 200 ms. A blank screen appeared for 500 ms followed by the motion stimulus for a further 500 ms, before being replaced by a black screen. Coherent dot direction could be in one of four directions (up, down, left, right) and participants were required to indicate via a key press the perceived direction of coherent dots in a four-alternate forced-choice design. The level of coherence was adjusted in a staircase procedure, which terminated after 10 reversals. Step size was 5%, and then 3% after the first reversal. The threshold coherence level was taken as the mean of the final 6 reversals.

#### Abrupt and Ramped Onset Object Recognition Tasks

One of eight line drawings of easily recognizable objects (e.g., clock, iron, teacup) were presented to participants in the middle of the screen, subtending between 8-10° by 8-10°. In the Abrupt contrast onset/offset task objects appeared at a constant contrast between foreground and background for four computer-refresh frames (50 ms). In the Ramped contrast onset/offset task, object contrast was ramped in a linear fashion, increasing from 0% to a maximum (over 13 frames), before reducing back to 0% contrast (over 13 frames), giving a total object duration of 325 ms. In both tasks, target objects were replaced by a rectangular white-noise mask (subtending 9° by 9°). Participants were then presented with four objects (the target, and three distracter line drawing objects), and were asked to identify with a keyboard press the target object, in a four-alternate force-choice match-to-sample paradigm. All distracters also appeared as targets in other trials, and no feedback was provided to the participant during the task.

Threshold contrast was determined by use of a staircase procedure by adjusting the percent contrast. The staircase terminated after 10 reversals and threshold was taken as the mean contrast level from the final 6 reversals. The eight objects were split into two halves with a separate staircase procedure conducted on each group of objects. This meant that within each staircase only one of four possible objects was presented, with the same four options (target and three distracters) always presented. The two staircase procedures were interwoven with each other such that each of the eight objects was presented in random order. The mean of the two resulting thresholds was used for data analysis. Inspection of raw data indicated that no participants reached a ceiling level, performing well below the lowest possible contrast.

### Procedure

Participants first completed the Ravens task, followed by, in counterbalanced order, the Motion Coherence detection and two Object Recognition tasks. Participants also completed a reading test and another visual psychophysics task as part of another project. The entire testing session duration was 1 hour. During the visual tasks participants sat in a darkened room. The visual task order was randomised between participants. Before each task began, an experimenter explained the task and gave demonstrations of trials until the participant understood the task.

## Results

Object recognition contrast thresholds from 5 participants were unreliable due to failed staircase procedures, with a further participant showing inconsistent motion coherence thresholds in repeated runs. These 6 participants were thus excluded and reduced our sample to 56.

**Figure 1:**
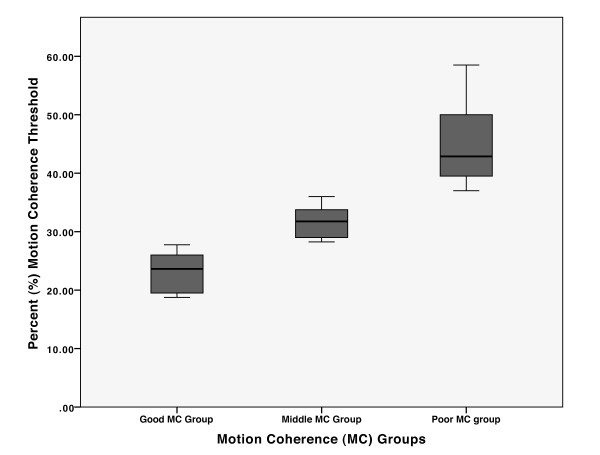
**Motion coherence detection thresholds for different Motion Coherence (MC) groups**. Box plots showing the distribution of the three motion coherence groups, created by taking the top, middle and bottom third of ranked threshold scores.

An initial correlation analysis demonstrated a poor linear relationship between Motion Coherence and Abrupt Object Recognition (*r *= .006, *p *= .96), and also between Motion Coherence and Ramped Object Recognition (*r *= -.119, *p *= .37). Given the nonlinear relationship between Motion Coherence and Abrupt Object recognition, participant thresholds for the Motion Coherence task were ranked, and the top and bottom thirds were categorized as the Good and Poor Motion Coherence (MC) groups, respectively. As is seen in Figure 1, this method was successful in creating two distinct subgroups, which differed significantly in their motion coherence abilities (Good MC: mean threshold = 23%, *n *= 18, CI [22,25]; Poor MC: mean threshold = 47%, *n *= 18, CI [43,50], *t*(36) = 12.1, *p *< .0001). These thresholds are slightly higher than previously found [33], though this is likely to be explained by the short duration of the stimulus (500 ms) and the limited lifetime of the dots (200 ms) giving a threshold in accordance with the results from Sutherland and Crewther [29] who used more similar parameters.

In order to test whether Good and Poor MC groups differed in some attribute other than motion coherence detection, groups were compared on the Ravens Progressive Matrices, and demonstrated no significant difference (*t*(34) = 0.18, *p *= .86).

**Figure 2:**
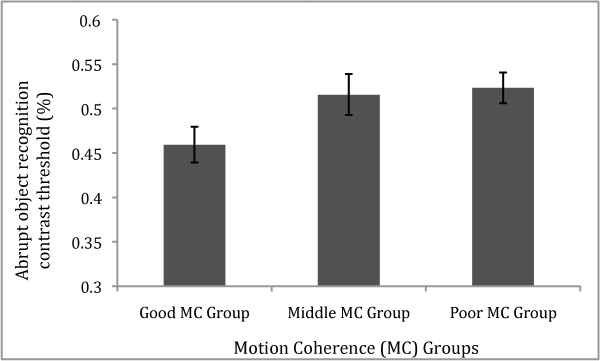
**Motion Coherence (MC) groups' Abrupt object recognition performance**. Bar graph shows mean contrast threshold (± SE) for the Good compared with the Poor MC groups, and the Middle MC group also shown, on the Object recognition task with abrupt presentation. The Good MC group showed significantly superior contrast threshold than the Poor MC group (*p *< .05).

**Figure 3:**
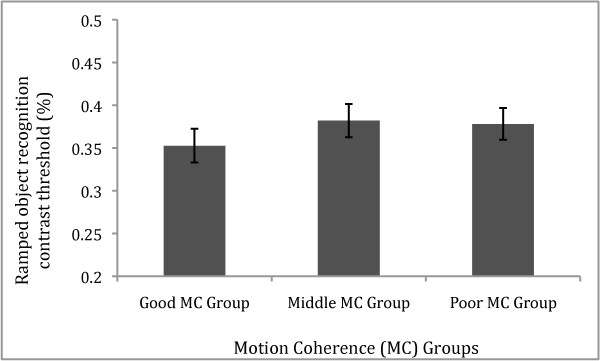
**Motion Coherence (MC) groups' Ramped object recognition performance**. Bar graph shows mean contrast threshold (± SE) for the Good compared with the Poor MC groups, and the Middle MC group also shown, on the Object recognition task with ramped presentation. The Good and Poor MC groups demonstrated equal contrast threshold (*p *> .05).

Finally, we compared the two subgroups of Motion Coherence abilities on the Abrupt and Ramped Object Recognition tasks. Figure 2 illustrates that the Good MC group demonstrated a lower mean contrast threshold than the Poor MC group on the Abrupt contrast onset/offset task. A t-test confirmed that the two groups differed significantly on the Abrupt task (*t*(34) = 2.10, *p *= .04, d = 0.70). Figure 2 also shows that the performance of the middle MC group is comparable with the Poor MC group (*p *= .82) On the other hand, as can be seen in Figure 3, Good and Poor MC groups showed very similar contrast thresholds for the Ramped contrast onset/offset task, with a t-test comparing groups showing no significant difference (*t*(34) = 0.76, *p *= .45). Similarly, Figure 3 also shows the performance of the middle MC group is comparable with the Poor MC group (*p *= .91)

## Discussion

The current study investigated the contribution of dorsal stream functioning in object recognition. This was achieved by manipulating the degree to which line-drawings of objects appeared suddenly or not by including abrupt and ramped onset tasks, and comparing two subgroups presumed to differ only in their performance on motion coherence ability - a task considered to be representative of dorsal stream functioning.

The two subgroups of motion coherence ability were only found to differ on an object recognition task when the objects had a relatively stronger attention-grabbing sudden appearance (i.e., objects appeared abruptly), and presumed to therefore more strongly activate bottom-up attention mechanisms in parietal cortex. It is suggested however, that Good and Poor MC groups did not differ in object specific processing *per se*. When the transient nature of the onset/offset of the object was reduced, by gradually ramping the contrast between foreground and background, the good and poor motion coherence groups showed comparable performance. We propose that the only difference between the two object recognition tasks relates to the physical presentation of the objects. In particular, our tasks differed in two ways: the nature of the onset, and the duration of the presentation of the target object (see further discussion of this below) rather than the objects themselves.

Given that the two subgroups showed equal nonverbal mentation, and therefore no differences in general attention or motivation, but differed only in their motion coherence ability, we suggest that relatively reduced dorsal stream functioning may help explain the differential object recognition performance with abrupt compared with ramped onset/offset stimuli. This would appear consistent with Levy et al. [30] who showed that good motion coherence detectors were better at classifying words as real words rather than nonwords compared with poor motion coherence detectors. In both Levy et al. and in the current study, skills known to require activation of ventral extrastriate cortex (reading and object recognition, respectively) were found to be related to proficiency in a known dorsal stream task (detection of motion coherence).

A possible explanation for the current findings relies on the magnocellular advantage model of visual processing [16] in which the rapid onset of stimuli is proposed to activate the largely magnocellular dorsal visual stream and to initiate visual attention mechanisms in parietal cortex. Rapid feedback to primary visual cortex, but potentially also horizontal connections from dorsal to ventral regions, or parietal to frontal connections followed by feedback to ventral stream regions may all contribute to a detailed analysis of the visual scene. The key aspect of this model for the current study relates to the rapid activation of parietal attention mechanisms by V5 and the dorsal stream - which are predicted to not be activated for visual stimuli with reduced salience (i.e., the ramped presentation in this study).

Such a model is supported by the work of Bar and colleagues who have used MEG to show that low spatial frequency visual information (i.e., magnocellular-type information) is projected through the dorsal stream to reach orbitofrontal cortex by approximately 130 ms, and then fusiform gyrus in the ventral stream 50 ms later [34]. Bar et al. argued that the dorsal stream projection to parietal and frontal cortices provides a course representation of the object, and triggers a top-down facilitation of detailed object processing into temporal cortex. This is similar to an "attentional spotlight" model utilising parietal mechanisms to guide temporal cortex processing [35]. However this model focuses more on spatial attention shifts, whereas the magnocellular advantage model [16] allows for rapid activation of attention directed to objects within central fixation well-suited to the magnocellular system (i.e., low spatial frequency, flickering, rapid or moving salient stimuli) to be used to initiate attention and/or higher cognitive processes and to facilitate later detailed processing in temporal cortex.

Given that the stimuli in the two tasks differed in the total duration (50 ms and 325 ms for abrupt and ramped objects, respectively), an alternative explanation may be posited. Rather than the presence or absence of an abrupt onset/offset activating the dorsal stream to initiate parietal attention mechanisms, the results may have more to do with difference between stimuli in temporal frequencies. It is possible that the abrupt stimuli were more efficient than ramped stimuli at activating high temporal frequency processing, likely to be handled by the magnocellular pathway. Thus, it may be that the abrupt stimuli activate a fast magnocellular response (in the Good MC, but not the Poor MC group), which is most likely fed through the dorsal stream [1,4]. On the other hand, the ramped task, consisting of the longer duration, and thus creating a slower temporal frequency would be less suited to activating early magnocellular processing in LGN. Such stimuli might be expected to rely less on the magnocellular advantage, with both MC groups therefore having to rely primarily on ventral stream processing.

The finding of a role for the dorsal stream in object recognition requires mention of previous experiments suggesting that the dorsal stream is involved in processing of specific categories of objects. Goodale and Milner [5,12] have proposed a dichotomy whereby the ventral stream handles vision for perception, whilst the dorsal stream is a non-conscious vision for action pathway. Fang and He [36] have shown with fMRI that the dorsal stream (functionally defined by the authors as areas corresponding to V3a/V7) still responded to a diverse range of object images rendered invisible by interocular suppression, but this effect was stronger in "tools" than faces. Other findings using similar paradigms have suggested that the dorsal stream influences ventral stream processing of manipulable objects (e.g., tools) [37,38].

Although most of the objects used in the current study might be considered manipulable (e.g., iron, tea-cup), we suggest that our results cannot be interpreted as evidence for dorsal stream involvement in the recognition of objects, which are manipulable as compared to other non-manipulable objects (e.g., words), and therefore cannot address the vision for action theory directly. This is due to the finding that although our participant groups (split on a measure of dorsal stream processing) differed on a measure of object recognition (of potentially manipulable objects) they did not show different performance when we adjusted the presentation format, but used the exact same objects. That is, it does not appear that the influence of dorsal stream ability on object recognition is related to the type of object, but is more likely related to the visual attributes (i.e., the nature of the onset/offsets) of these objects.

Furthermore, there is a large literature that has argued that the dorsal stream is involved in object recognition unrelated to action or the manipulation of objects. For example previous work has demonstrated dorsal stream involvement in word recognition [13,27,30,34]. The likely role of the dorsal stream in this type of non-manipulable object processing is likely to be in initiating frontoparietal attention mechanisms and in facilitating top-down facilitation of ventral stream object processing. Bar et al [34], for example, provided MEG evidence that an early magnocellular projection through the dorsal stream activated orbitofrontal cortex and was followed by a later ventral stream (fusiform) activation for objects (e.g., tools, furniture, clothes, animals, means of transportation).

## Conclusions

The current data cannot establish a causal relationship between dorsal stream functioning (as assessed by motion coherence performance) and relatively more or less transient onset/offset object recognition. As alluded to earlier Laycock et al. [13] used TMS to disrupt V1/V2 and V5 in a word recognition task. Reduced single (abrupt onset) word identification following TMS induced disruption of V5 argues for a causal role of the dorsal stream in rapid accurate (fluent) reading. TMS provides the opportunity to further explore the *necessary *role of dorsal and ventral visual regions in object recognition while allowing the mapping of the temporal order of events within early dorsal, parietal and ventral regions. Nevertheless, this psychophysical experiment has produced further evidence that the dorsal stream is required for detection of abrupt but not for ramped onset/offset objects discrimination.

## Competing interests

The authors declare that they have no competing interests.

## Authors' contributions

RL conceived of the study and participated in it's design and coordination, designed visual stimuli, and helped to draft the manuscript. AJC helped with study design and data collection. TL helped with study design and data collection. SGC conceived of the study, and participated in its design, and helped to draft the manuscript. All authors read and approved the manuscript.
